# HDL-Associated Specific Paraoxonase-1 Activity Is Linked to Atherogenic Lipoprotein Measures in a High Cardiovascular Risk Population: A Cross-Sectional Study

**DOI:** 10.3390/antiox15060731

**Published:** 2026-06-09

**Authors:** Linas Černiauskas, Viktorija Palšytė, Goda Aleknavičiūtė, Eglė Mazgelytė, Inga Bikulčienė, Jolita Badarienė, Egidija Rinkūnienė, Skaistė Arbačiauskaitė, Susann Allelein, Dovilė Karčiauskaitė

**Affiliations:** 1 Department of Physiology, Biochemistry, Microbiology and Laboratory Medicine, Institute of Biomedical Sciences, Faculty of Medicine, Vilnius University, M. K. Čiurlionio st. 21, LT-03101 Vilnius, Lithuania; 2 Biomarker Research Laboratory, Translational Health Research Institute, Faculty of Medicine, Vilnius University, Žaliųjų ežerų st. 2, LT-08406 Vilnius, Lithuania; 3 Department of Preclinical Research, Center for Innovative Medicine, Santariškių st. 5, LT-08406 Vilnius, Lithuania; 4 Clinic of Cardiac and Vascular Diseases, Institute of Clinical Medicine, Faculty of Medicine, Vilnius University, M. K. Čiurlionio st. 21, LT-03101 Vilnius, Lithuania; 5 Neuroscience Research Center, Charité-Universitätsmedizin Berlin, 10117 Berlin, Germany; 6 MicroDiagnostics Unit, Fraunhofer Institute for Cell Therapy and Immunology IZI, 04103 Leipzig, Germany

**Keywords:** paraoxonase 1, high-density lipoproteins, apolipoprotein B, lipoprotein(a), cardiovascular diseases, ultracentrifugation

## Abstract

Despite increasing efforts to improve cardiovascular disease (CVD) risk evaluation and management, it remains a leading cause of mortality and morbidity worldwide. This has driven interest in high-density lipoprotein (HDL)-related biomarkers as indicators of oxidative stress and atherogenic processes not fully captured by traditional lipid measurements. In this study, we examined specific paraoxonase 1 (PON1) activity and its relationship with anthropometric, blood pressure, and lipid metabolism measures in 100 middle-aged Lithuanian individuals at high cardiovascular risk. HDL fractions were isolated using iodixanol-based density gradient centrifugation. PON1 concentration and arylesterase activity were measured, and specific activity was defined as arylesterase activity normalized to PON1 concentration. No significant associations were observed between specific PON1 activity and age, body mass index, waist circumference, blood pressure, smoking status, or statin use. Specific PON1 activity was independently associated with lower risk-weighted apolipoprotein B and lower low-density lipoprotein cholesterol levels. These exploratory findings suggest that higher specific PON1 activity may reflect a less atherogenic lipid profile in individuals at high cardiovascular risk, as indicated by its association with LDL-C and with risk-weighted apolipoprotein B. Because direct oxidative stress and inflammatory markers were not measured, interpretations regarding oxidative burden should be considered indirect and hypothesis-generating. Given the cross-sectional nature of the study and the relatively small sample size, these results should be interpreted as exploratory and hypothesis-generating. Further longitudinal studies in larger populations are needed to confirm these observations.

## 1. Introduction

Cardiovascular disease (CVD) remains the leading cause of mortality worldwide, with a particularly high burden in Eastern European regions [1]. In Lithuania, which is categorized as a very high cardiovascular risk country, CVD accounted for more than half (50.8%) of all deaths in 2024 [2,3]. Many cardiovascular diseases are primarily driven by atherosclerosis, a lipid-driven inflammatory disease closely associated with lipid oxidation and oxidative stress [4]. Currently, traditional lipid metabolism markers, such as low-density lipoprotein cholesterol (LDL-C) and non-high-density lipoprotein cholesterol (Non-HDL-C), do not capture the oxidative component driving atherosclerosis. This leads to growing interest in other lipoprotein-related biomarkers and their combinations that may better reflect atherosclerosis [5]. Apolipoprotein B (Apo B) is increasingly recognized as a superior marker of atherogenic particle burden compared with cholesterol-based measures [5,6]. More recently, composite metrics incorporating lipoprotein(a) (Lp(a)) have been proposed to capture residual risk better, as Lp(a) carries oxidized phospholipids and confers increased atherogenicity per particle [7,8,9]. Risk-weighted Apo B (Rw Apo B), which integrates Apo B and Lp(a), may therefore better reflect the qualitative atherogenic potential and partially capture the oxidative component of circulating lipoproteins [8].

Additionally, over the last two decades, the high-density lipoprotein (HDL) hypothesis has been disproven by the failure of high-density lipoprotein-cholesterol (HDL-C)–raising therapies to reduce cardiovascular mortality, as well as by results from Mendelian randomization studies [10,11,12]. This shifted interest from HDL quantity as the sole metric in cardiovascular disease estimation to research aimed at assessing other structural and functional components of HDL particles [13]. HDL antioxidant enzymes such as paraoxonase 1 (PON1) are now considered essential contributors to HDL’s atheroprotective effects, and their measurement as a proxy for HDL function may provide more relevant insights into cardiovascular risk than HDL particle number metrics alone [13,14]. In addition, PON1 arylesterase activity is suggested to play a key role in hydrolyzing oxidized lipids in Apo B–containing lipoproteins, adding to its potential role in reducing oxidation-related atherogenic burden [15]. Furthermore, PON1 arylesterase activity is proposed to be a stable indicator of PON1 function, as it is least influenced by genetic polymorphisms among its other enzymatic activities, making it useful for biomedical studies [15,16,17]. Additionally, reduced PON1 activity has been linked to cardiovascular disease and metabolic disorders, suggesting that enzyme activity, rather than concentration, better reflects its protective role [15,18]. However, accurate assessment of PON1 activity requires preservation of its native association with HDL, as it is strongly dependent on lipoprotein interactions [19,20]. In this context, methodological approaches that preserve physiological HDL structure are essential for estimating HDL particle functionality in vitro. Recently, methods for HDL isolation, such as density gradient centrifugation (DGC) using iodixanol, an iso-osmotic, non-destructive ultracentrifugation medium, have emerged as a less disruptive alternative to traditional potassium bromide (KBr)–based ultracentrifugation methods. These methods have been shown to maintain native lipid–protein interactions, which are crucial for PON1 activity, and to enable a more physiologically relevant assessment of HDL-associated proteins [19,20,21,22]. However, studies using this approach for lipoprotein fractionation and PON1 activity estimation are very scarce, thus limiting knowledge of the applicability and usefulness of these methods in HDL-related cardiovascular research.

Therefore, this study aimed to evaluate specific PON1 activity in HDL-associated PON1-rich fractions after density-gradient ultracentrifugation in 100 middle-aged individuals with high cardiovascular risk. In addition, the study aimed to evaluate the relationship between specific PON1 activity and anthropometric characteristics, lipid metabolism measures, and the novel composite marker Rw Apo B in high cardiovascular risk individuals to better understand its role in cardiovascular disease.

## 2. Materials and Methods

### 2.1. Study Participants and Sample Collection

The participants were recruited consecutively from individuals attending the Lithuanian national cardiovascular prevention program at Vilnius University Hospital Santaros Clinics, Center of Cardiology and Angiology, between April and June 2024. Participants were enrolled consecutively; all eligible individuals who attended the center during the recruitment period, met the inclusion criteria, and provided written informed consent were enrolled. No random sampling procedure was performed. The inclusion criteria were age ≥40 to ≤65 years and participation in the national cardiovascular prevention program in Lithuania. Exclusion criteria were age <40 or >66 years, individuals not participating in the national cardiovascular disease prevention program, pregnant or breastfeeding individuals. All participants provided written informed consent before inclusion in the study. The study protocol was approved by the Vilnius Regional Biomedical Research Ethics Committee (No. 2023/9-1518-998), and all procedures comply with the tenets of the Declaration of Helsinki.

Venous blood samples were collected from participants after an overnight fast by trained staff using BD Vacutainer™ tubes (BDFranklin Lakes, NJ, USA). For plasma preparation, two 6 mL lithium heparin tubes (17 IU/mL) were collected from each participant, along with one serum separator tube (BD Vacutainer™ SST™, clot activator with polymer gel, 8.5 mL).

The heparin tubes were gently inverted 8–10 times after collection to ensure proper mixing with the anticoagulant and were processed within 2 h of venipuncture. Plasma was obtained by centrifugation at 2500× *g* for 15 min at room temperature. The upper plasma layer was carefully removed, avoiding the buffy coat, transferred to a clean microcentrifuge tube, and centrifuged again under the same conditions to obtain platelet-free plasma, following recommendations of the International Society on Thrombosis and Haemostasis (ISTH) [23]. The final supernatant was aliquoted into 0.5 mL portions in safe-lock microcentrifuge tubes and stored at −80 °C until analysis.

Serum samples were allowed to clot for 30 min at room temperature and then centrifuged at 2000× *g* for 10 min at room temperature. The resulting serum was aliquoted into 0.5 mL portions and stored at −80 °C until analysis.

### 2.2. Health-Related Data and Anthropometric Measure Collection

Health-related data, including age, sex, statin use, and smoking status, were obtained from electronic health records. Anthropometric measurements (height, weight, waist circumference) were collected by trained staff following standard procedures. Body mass index (BMI) was calculated according to the World Health Organization (WHO) definition as weight in kilograms divided by height in meters squared [24]. Resting systolic (SBP) and diastolic blood pressure (DBP) were measured on the dominant arm at heart level using an appropriately sized cuff, after the participant had been seated at rest for at least 5 min [25]. Mean arterial blood pressure (MABP) was calculated from these values as the diastolic blood pressure plus one-third of the difference between the systolic and diastolic blood pressures. Hypertension was defined according to the blood pressure guideline criteria as systolic blood pressure ≥140 mmHg and/or diastolic blood pressure ≥90 mmHg [25]. Metabolic syndrome was defined according to the National Cholesterol Education Program Adult Treatment Panel III (NCEP ATP III) criteria as the presence of at least three of the following: increased waist circumference, elevated triglycerides, reduced HDL-C, elevated blood pressure, and elevated fasting glucose or previously diagnosed diabetes mellitus [26]. Diabetes mellitus and family history of cardiovascular disease were determined from electronic health records.

### 2.3. Lipid Metabolism Parameter Measurements

Serum concentrations of total cholesterol, high-density lipoprotein cholesterol (HDL-C), and triglycerides (TG) were measured using routine enzymatic methods on the Architect ci8200 analytical system (Abbott, Abbott Park, IL, USA). Low-density lipoprotein cholesterol (LDL-C) was calculated using Friedewald’s equation for individuals with TG < 1.7 mmol/L. For those with TG ≥ 1.7 mmol/L, a direct LDL-C assay was used on the Architect ci8200 analytical system (Abbott, USA). Non–HDL-C was obtained by subtracting HDL-C from total cholesterol. Apolipoprotein B (Apo B) was measured by nephelometry on the Siemens BNII system (Siemens Medical Solutions USA, Inc., Malvern, PA, USA). Lp(a) concentrations were determined by immunoturbidimetry using the Optilite analytical system (The Binding Site Group Ltd., Birmingham, UK), and apolipoprotein A-I (Apo A-I) was measured by immunoturbidimetry on the Architect ci8200 analytical system (Abbott, USA). Risk-weighted Apo B (Rw Apo B) concentration was calculated as previously described by Rehman and Tudrej [8]. Apo B values measured in g/L were converted to nmol/L by multiplying by 2000, which is equivalent to the published conversion from mg/dL to nmol/L and assumes a molecular weight of 500 kDa. Rw Apo B was then derived as Apo B plus 6 × Lp(a), and results were expressed in µmol/L.

### 2.4. HDL-Associated Fraction Separation Using Density-Gradient Ultracentrifugation

Density gradient centrifugation was performed using a Sorvall WX+ Ultracentrifuge (Thermo Fisher Scientific, Waltham, MA, USA) equipped with a SureSpin 632 Rotor (Thermo Fisher Scientific, USA) and 17 mL polyallomer tubes (Seton Scientific Inc., Petaluma, CA, USA). Centrifugation was carried out at an average relative centrifugal force (RCF_avg_) of 119,800× *g* for 40 h at 4 °C. Four-step iodixanol gradients (10–40%) were prepared in 1× Tris-EDTA (TE) buffer (pH 7.4), with sucrose concentrations inversely adjusted to maintain consistent osmolality (208, 167, 125, and 83 mM). The gradients were layered sequentially from bottom to top, starting with 40% iodixanol and ending with 10%. Plasma samples (0.5 mL) were diluted 1:1 with 1 × phosphate-buffered saline (PBS) (filtered through a 0.22 µm filter) before loading. The diluted plasma was carefully layered onto the prepared gradients before ultracentrifugation. Following centrifugation, 16 density gradient fractions (F1–F16) were collected by sequentially aspirating 1 mL from the top to the bottom of the tube. Collected fractions were immediately frozen and stored at −80 °C until further analysis.

### 2.5. DGC Fraction Analysis

Apo A-I, Apo B, and PON1 concentrations in fractions F1–F16 were measured spectrophotometrically using commercial enzyme-linked immunosorbent assay (ELISA) kits (Apo A-I: E-EL-H0125; Apo B: E-EL-H6171; PON1: E-EL-H2298; Elabscience Biotechnology Inc., Houston, TX, USA). Total protein concentration was determined using a Thermo Scientific Pierce BCA protein assay (Thermo Fisher Scientific, USA). All analyses were conducted according to the manufacturer’s instructions.

### 2.6. PON1 Activity, and Specific Activity

PON1 arylesterase activity in fraction F4 was determined by measuring the hydrolysis of p-nitrophenyl acetate (pNPA) to p-nitrophenol (pNP), with some modifications as described by Tvarijonaviciute et al. [27]. The assay was performed in 96-well microplates with a total reaction volume of 200 µL, containing 2 µL of fraction F4, 2.5 mM p-nitrophenyl acetate, 2 mM CaCl_2_, and 50 mM Tris buffer (pH 8.0). The reaction was measured every 15 s at 405 nm for 6 min at 37 °C using a microplate spectrophotometer Infinite^®^ M Nano (Tecan Group Ltd., Männedorf, Switzerland). A blank sample was included to account for spontaneous substrate hydrolysis, and a pooled plasma sample was used as a positive control in each run. All sample measurements were performed in triplicate. PON1 activity was estimated from the linear phase of the enzymatic reaction (1–5 min) based on the change in absorbance over time (_Δ_OD), using the Beer–Lambert law with an extinction coefficient of 14,000 M^−1^·cm^−1^. The values were normalized to plasma volume and expressed as units per milliliter (U/mL). Specific PON1 activity was calculated by dividing the PON1 activity in F4 by the PON1 concentration in F4. Results were expressed as kU/mg of PON1.

### 2.7. Statistical Analysis

The sample size was determined pragmatically based on participant availability during the recruitment period and the resource-intensive nature of density-gradient ultracentrifugation analyses. No a priori sample size calculation was performed. A sensitivity analysis indicated that, with n = 100, α = 0.05, and 80% power, the study could detect approximately moderate effect sizes (e.g., correlation coefficient r ≈ 0.276), whereas smaller associations may have remained undetected. Tertile-based analyses, yielding 33 or 34 participants per group, were performed for descriptive purposes only, while continuous correlation and regression analyses were considered the primary inferential analyses. No missing data were present for variables included in the analyses.

The normality of continuous variables was assessed using the Shapiro–Wilk test. Normally distributed variables are presented as mean ± standard deviation (SD), while non-normally distributed variables are presented as median and interquartile range (IQR). Categorical variables are presented as counts and percentages. Comparisons between groups were performed according to data distribution and test assumptions. For normally distributed variables with homogeneity of variances, differences were evaluated using a one-way ANOVA followed by Tukey’s post hoc test. For non-normally distributed variables, differences across groups were evaluated using the Kruskal–Wallis test, followed by Dunn’s post hoc test with Benjamini–Hochberg FDR adjustment for multiple comparisons. A chi-square test was used to assess associations between categorical variables. In addition, linear trend analyses across tertiles were performed for continuous variables by coding tertile groups as an ordinal variable (0–2) and assessing ordered associations using linear regression. Associations between continuous variables were assessed using Spearman’s rank correlation test. The strength of Spearman’s rank correlations was interpreted as weak (|r_s_| < 0.30), moderate (|r_s_| 0.30–0.49), strong (|r_s_| 0.50–0.69), and very strong (|r_s_| ≥ 0.70) [28].

Multivariable linear regression was performed using the ordinary least squares method. Skewed variables were ln-transformed before analysis. Regression coefficients were estimated and reported with 95% confidence intervals, calculated using the Wald method. Regression assumptions, including linearity, normality of residuals, homoscedasticity, absence of multicollinearity, and independence of errors, were assessed using appropriate diagnostic tests and visual inspection. In models with violations of regression assumptions, HC3 robust standard errors were used to ensure valid inference. Four models were constructed for each dependent variable: Model 1 included only specific PON1 activity; Model 2 was additionally adjusted for sex and age; Model 3 was additionally adjusted for BMI; and Model 4 was further adjusted for statin use and smoking. Sex and age were included because they are essential demographic determinants of lipid metabolism and influence PON1 activity. BMI was included as an adiposity-related covariate, while smoking status and statin use were selected for their impact on PON1 activity and lipid metabolism. The primary multivariable models were restricted to a limited number of clinically relevant covariates to reduce the risk of model overfitting. Sensitivity analyses were additionally performed for the primary regression outcomes by repeating the fully adjusted models after inclusion of hypertension or metabolic syndrome as additional covariates. Exploratory interaction analyses were additionally performed in the fully adjusted models to assess whether associations between specific PON1 activity and the primary lipid metabolism-related outcomes differed by sex.

For pairwise comparisons following a one-way ANOVA, Tukey’s post hoc test was applied, which inherently accounts for multiple comparisons; therefore, these *p*-values were not adjusted using FDR. Where applicable, *p*-values were adjusted for multiple comparisons using the Benjamini–Hochberg false discovery rate (FDR) method and reported as q-values. A two-tailed *p*-value < 0.05 or q-value < 0.05, as appropriate, was considered statistically significant. All statistical analyses were conducted using R (v4.3.3; R Foundation for Statistical Computing, Vienna, Austria) and Jamovi (v2.7.23.0).

## 3. Results

### 3.1. Sample Characteristics

The study population (n = 100) showed an overall unfavorable cardiometabolic profile (Table 1). Participants were generally characterized by overweight or obesity, central adiposity, and elevated systolic and diastolic blood pressure. Atherogenic lipid measures were also elevated, notably LDL-C, non-HDL-C, and Apo B levels above recommended clinical targets [6]. Additionally, half of the participants were current smokers, and more than one-third were receiving statin therapy.

Taken together, these findings indicate that the study population had elevated cardiovascular risk based on current clinical guidelines [6,25].

### 3.2. Protein Distributions in DGC Fractions

**Figure 1:**
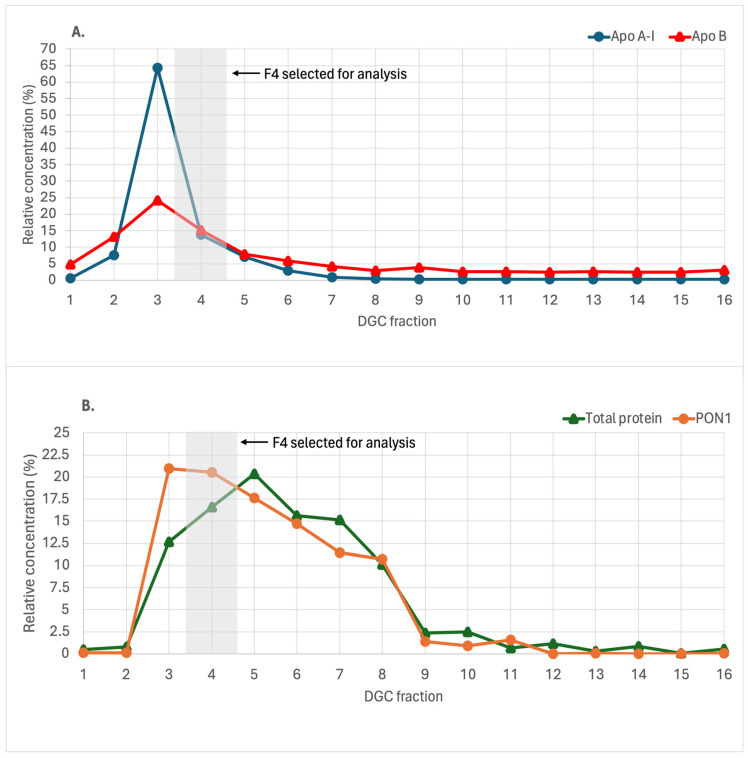
Protein distributions across DGC fractions 1 to 16. (**A**) Lipoprotein marker Apo A-I and Apo B distributions. (**B**) PON1 and total protein distributions.

Based on the DGC method, iodixanol density and gradient overlap during centrifugation, Apo A-I containing HDL would be expected to be present in F3–F12, while Apo B containing LDL in F1-F5. This is demonstrated by our results, where 98.87% of Apo A-I and 65.08% of Apo B are present in the corresponding DGC fractions. Furthermore, fraction F4 was selected for PON1 level and activity analysis as a practical balance between maintaining a high PON1 and reducing LDL-associated contamination. Although F3 showed the highest PON1 proportion (20.95%), it also had higher Apo B levels (24.16%), indicating greater contamination with non-HDL particles. In contrast, F4 retained a comparable PON1 level (20.51%) while showing lower Apo B content (15.15%) (Figure 1).

F4 contained detectable Apo A-I (13.71%), suggesting that PON1 remained associated with HDL particles, although to a lesser extent than in F3. In addition, the slightly higher total protein content in F4 (16.58% vs. 12.67%) provided sufficient material for downstream analyses of PON1 enzyme activity.

### 3.3. Clinical Variables Across Specific PON1 Activity Tertiles

Clinical variables, including age, sex distribution, anthropometric measures, blood pressure, lipid metabolism parameters, smoking status, and statin use, did not differ statistically significantly across PON1 activity tertiles (Table 2). A difference observed among Rw Apo B medians among specific PON1 activity tertiles did not remain statistically significant after FDR adjustment. PON1 levels and PON1 activity statistically significantly differed among specific PON1 activity tertiles, as expected, because specific PON1 activity was derived from PON1 activity and PON1 concentration. Finally, the correlation analysis between PON1 levels and PON1 activity revealed a moderate negative association (rs = −0.346, q = <0.001).

### 3.4. Association Between Specific PON1 Activity and Demographic, Anthropometric, and Blood Pressure Measures

Correlation analysis revealed only weak negative associations between specific PON1 activity and demographic, anthropometric, and blood pressure variables. However, none of these associations were statistically significant before and after FDR adjustment (Table 3).

### 3.5. Association Between Specific PON1 Activity and Lipid Metabolism Parameters

The further correlation analysis revealed mainly weak negative associations between specific PON1 activity and lipid metabolism parameters, except for HDL-C, triglycerides, and Apo A-I, for which weak positive associations were observed (Table 4).

Statistically significant results were observed between specific PON1 activity and LDL-C (r_s_ = −0.225, *p* = 0.025), Apo B/Apo A-I (r_s_ = −0.240, *p* = 0.016), and Rw Apo B values (r_s_ = −0.287, *p* = 0.004). However, after FDR adjustment, only the weak negative association between specific PON1 activity and Rw Apo B remained statistically significant (r_s_ = −0.287, q = 0.040).

### 3.6. Specific PON1 Activity Association with Atherogenic Lipid Metabolism Measures

For regression analysis, Lp(a) and Rw Apo B were ln-transformed, and coefficients represent proportional (percentage) changes in the dependent variables (Table 5).

Specific PON1 activity was inversely associated with LDL-C and ln (Rw Apo B) across all models and remained statistically significant after full adjustment (LDL-C: β = −0.041, 95% CI [−0.071; −0.010], *p* = 0.010, q = 0.040); (ln (Rw Apo B): β = −0.012, 95% CI [−0.021; −0.004], *p* = 0.006, q = 0.034). Practically, this corresponds to a decrease of approximately 0.041 mmol/L in LDL-C and a 1.2% reduction in Rw Apo B per 1 kU/mg increase in specific PON1 activity. The fully adjusted models were statistically significant for both LDL-C (*p* < 0.001) and ln (Rw Apo B) (*p* = 0.013) and explained 23% of the variance in LDL-C and 10% of the variance in ln (Rw Apo B). In addition, sensitivity analyses additionally adjusting for hypertension or metabolic syndrome did not alter the direction or magnitude of the observed associations between specific PON1 activity and LDL-C and ln (Rw Apo B) (Table S2). In particular, the β coefficients and their confidence intervals remained similar to those observed in the primary analyses, and the associations remained statistically significant after FDR correction. Exploratory interaction analyses in the fully adjusted models did not demonstrate statistically significant sex and specific PON1 activity interactions for LDL-C (*p* = 0.181) or ln (Rw Apo B) (*p* = 0.208), suggesting that the direction of the associations was similar between men and women. Inverse associations with Apo B/Apo A-I were observed in unadjusted and adjusted for age and sex models, but were attenuated after full adjustment and were not significant after FDR correction. Associations with Apo B and non-HDL-C were not statistically significant. Although associations with ln (Lp(a)) were observed in some models, including the fully adjusted model, only the unadjusted model was statistically significant (Table S1).

## 4. Discussion

In this study, DGC using a non-destructive, iso-osmotic gradient was applied to fractionate lipoproteins and select a PON1-rich HDL-associated fraction for analysis. In contrast to traditional KBr-based ultracentrifugation methods, which may alter lipoprotein composition due to high ionic strength and osmotic stress, iodixanol-based gradients are considered less disruptive and may better preserve lipoprotein particle integrity and HDL-associated proteins [22,29,30]. Some studies have shown that salt-based ultracentrifugation can disrupt lipoprotein structure, whereas iodixanol maintains lipid–protein interactions and reduces structural alterations [21,22]. The main drawback is that using iodixanol gradients reduces separation efficiency, so the resulting fractions are better described as only HDL-associated fractions rather than distinct, pure HDL subfractions [31]. In this context, the choice of fraction for PON1 analysis may need to consider not only the highest PON1 signal but also minimizing contamination from Apo B-containing lipoproteins while maintaining a substantial Apo A-I concentration. In this study, the fraction F3 with the highest PON1 levels was associated with higher Apo B levels than the fraction F4, which was selected for PON1 analysis. This suggests co-isolation of non-HDL particles and indicates that the fraction with the greatest PON1 abundance is not necessarily the most suitable for analysis, as some particles containing Apo B, notably chylomicrons and very low-density lipoproteins, have been shown to exhibit some PON1 activity [32]. Because F4 still contained detectable Apo B, the measured activity should be interpreted as activity in an HDL-associated, PON1-rich fraction rather than in a completely purified HDL fraction. In addition, fractions with lower Apo B content may provide a less Apo B-contaminated estimate of HDL-associated PON1 activity. The presence of Apo A-I in these fractions is consistent with the presence of HDL-associated PON1, although the fraction was not a pure HDL subfraction. These findings are consistent with previous reports showing that PON1 is distributed across different HDL particles rather than confined to a single HDL subclass [33,34]. Earlier studies, particularly those using salt-based ultracentrifugation methods, often described enrichment of PON1 in dense HDL3 fractions; however, under conditions that better preserve native structure, the distribution appears broader and reflects native HDL heterogeneity [34,35]. Importantly, measuring PON1 in these fractions may provide a more physiologically relevant estimate than measurements after extensive purification, because HDL-associated interactions are partly preserved. PON1 activity depends on its association with HDL, especially interactions with Apo A-I and phospholipids that stabilize the enzyme and support its catalytic function [19,20]. Previous studies suggest that disruption of these interactions can reduce PON1 activity, making isolated measurements less reflective of physiological conditions [36]. Overall, these results suggest that iodixanol-based DGC provides a useful approach for studying PON1 under conditions that may better preserve its native environment. At the same time, they highlight the importance of selecting fractions with minimal Apo B contamination while maintaining substantial Apo A-I levels to increase the likelihood that the measured activity predominantly reflects HDL-associated PON1 rather than dissociated or freely circulating enzyme.

The analysis of PON1 concentration and activity revealed a modest inverse correlation between PON1 concentration and PON1 activity, suggesting PON1 concentration did not directly reflect arylesterase activity in the analyzed fraction. Comparison of this association across studies is difficult due to the lack of standardized PON1 evaluation techniques [17]. For instance, there is a significant discrepancy in the samples used to evaluate PON1 activity: some studies use whole serum or plasma, while others fractionate plasma or serum using salt-based ultracentrifugation, chemical precipitation, or electrophoretic methods [37,38,39,40]. The choice of sample has been shown to affect PON1 activity measures [17]. In addition, PON1 has three predominant enzymatic activities: lactonase, paraoxonase, and arylesterase [17,37]. This also causes significant inconsistency across studies, as the choice of assay is tailored to specific enzymatic activity, which is suggested to differ across PON1 genotypes [15,41]. For example, the *Q192R* polymorphism has substrate-dependent effects and more strongly affects other PON1 activities than phenyl acetate-based arylesterase activity, whereas *L55M* and the promoter variant *-108C/T* may influence PON1 expression, protein stability, or activity. Moreover, PON1 arylesterase activity in HDL subfractions has been reported to differ according to *Q192R* genotype, suggesting that unmeasured PON1 genetic variation may contribute to between-study variability in arylesterase activity estimates [38]. The findings of this study indicate that higher PON1 mass does not necessarily translate into increased arylesterase enzymatic activity. Furthermore, factors such as sample type, the choice of substrate, and genotype variations are important for assessing function in PON1 studies and ensuring comparability.

Recent studies suggest that sex-related differences are relevant when interpreting PON1 activity [42,43]. Trentini et al. reported that serum PON1 arylesterase and lactonase activities were higher in women than in men, independently of HDL-C, age, smoking status, BMI, and waist circumference [42]. Similarly, Rosta et al. showed that the reduction in serum PON1 arylesterase activity associated with type 2 diabetes was more pronounced in women than in men, suggesting that PON1-related activity may be influenced by sex in metabolic disease [43]. However, sex-related differences in PON1 activity are not uniform across populations. Meisinger et al. found in the population-based study that sex did not significantly modify the associations between inflammatory markers and serum PON1 arylesterase activity [44]. In the present cohort, sex distribution did not differ significantly across specific PON1 activity tertiles, and exploratory interaction analyses did not show a statistically significant modification by sex for the associations of specific PON1 activity with LDL-C or ln (Rw Apo B). These exploratory findings suggest that the observed associations between specific PON1 activity and atherogenic lipid measures were broadly similar in women and men in this study. Nevertheless, because the study was not powered for detailed sex-stratified analyses and did not include hormonal or menopausal status, smaller sex-specific effects cannot be excluded and should be examined in larger studies.

Furthermore, in this study, specific PON1 activity was not associated with age, anthropometric measures, or blood pressure. This interpretation is consistent with the literature, which indicates that PON1 activity is influenced by genetic variation, HDL-related factors, oxidative status, and inflammation rather than by anthropometric indices alone [15,42,45,46,47,48]. However, the evidence remains contradictory, as some studies have reported associations between PON1 activity, age, BMI, and blood pressure [49,50]. In addition, lower PON1 activity is associated with metabolic syndrome, obesity-related states, and other conditions, including hypertension [40,41,43,51]. These discrepancies among study findings may partially reflect that in the present study, higher PON1 mass did not necessarily correspond to higher arylesterase enzymatic activity. differences in population characteristics, the substrate and sample choice for the PON1 assay, the distribution of PON1 genotypes, and whether absolute or specific activity was evaluated [17].

In the present cross-sectional analysis, higher specific PON1 activity was independently associated with selected atherogenic lipid measures, particularly lower LDL_C and ln (Rw Apo B). This is demonstrated by inverse associations between specific PON1 activity and LDL-C, and between PON1 activity and ln (Rw Apo B)—a composite metric integrating Apo B particle number with the additional atherogenic contribution of Lp(a) [8]. The persistence of the association with ln (Rw Apo B) across models suggests that specific PON1 activity may be related to composite Apo B/Lp(a)-based lipid burden rather than Apo B concentrations alone. One possible biological explanation, based on previous literature, is that PON1 protects Apo B-containing lipoproteins from oxidative modification [15]. Rw Apo B captures not only the number of atherogenic particles but also Lp(a), which has been shown to contain oxidized phospholipids and confers high cardiovascular risk per particle [7,9,52]. The observed inverse relationship between specific PON1 activity and ln (Rw Apo B), along with the inconsistent relationship with ln (Lp(a)), is compatible with the hypothesis that HDL-associated PON1 activity may be related to oxidative processes involving Lp(a) and Apo B-containing particles, although oxidative burden was not directly measured in this study [20]. Interestingly, the attenuation of associations with Apo B suggests that Apo B concentration alone may not capture the same information as the composite Rw Apo B measure. While Apo B reflects particle number, it does not account for oxidative modification, which is a key factor in lipoprotein pathogenicity. Previous studies indicate that oxidative changes in Apo B–containing lipoproteins may contribute to atherogenesis independently of particle concentration [50,51,52]. In this context, the stronger and more consistent association observed with ln (Rw Apo B) supports further investigation of composite lipid measures in relation to HDL-associated PON1 function.

The inverse association between specific PON1 activity and LDL-C should be interpreted in the context of Apo B–containing lipoprotein biology and the limitations of currently used LDL-C measurement methods. LDL-C primarily reflects cholesterol within LDL particles but also includes some of the cholesterol content of other Apo B–containing lipoproteins, including Lp(a) and intermediate density lipoproteins [53]. This limitation is particularly relevant when LDL-C is estimated using the Friedewald equation or standard direct assays, both of which are susceptible to inaccuracies and incomplete separation of lipoprotein fractions [54]. Consequently, LDL-C in this setting should be considered as a proxy measurement for cholesterol within atherogenic Apo B–containing particles rather than a specific measure of LDL cholesterol alone. Given that PON1 is suggested to exert antioxidant effects across Apo B–containing lipoproteins, including LDL and Lp(a), the observed inverse association with LDL-C may reflect an association between specific PON1 activity and atherogenic lipoprotein burden, but whether this relates to oxidative burden requires direct testing. This interpretation is supported by evidence that PON1 protects lipoproteins from oxidative modification while oxidative stress can impair specific PON1 activity, suggesting a potential bidirectional effect [15,55]. The stronger and more consistent association observed with ln (Rw Apo B) further supports the concept that PON1 activity is more closely related to the cumulative atherogenic potential of circulating lipoproteins than to LDL-C alone. An apparent discrepancy was observed between LDL-C, which showed an inverse association with specific PON1 activity, and non-HDL-C, which did not. Although both are cholesterol-based measures, LDL-C is more closely related to lipoprotein fractions that are especially susceptible to oxidation, such as small dense LDL, and may reflect oxidative processes potentially related to PON1 activity [56,57]. In contrast, non-HDL-C represents the total cholesterol content across a broader, more heterogeneous pool of Apo B–containing lipoproteins than LDL-C, which differ substantially in composition, cholesterol content, and susceptibility to oxidation [58]. As a result, non-HDL-C may function as a measure of total cholesterol in Apo B-containing atherogenic lipoprotein particles rather than reflect oxidized lipoproteins. In addition, non-HDL-C is a calculated parameter derived from total cholesterol and HDL-C and incorporates variability from both components. This may introduce additional complexity in interpretation, as HDL-C is closely linked to PON1 biology, given that PON1 is associated with HDL particles and is a measure of their functionality. As a result, including HDL-C in the non-HDL-C calculation may introduce opposing or confounding factors that attenuate associations with specific PON1 activity. Given that specific PON1 activity is biologically linked to the oxidative modification of lipoproteins rather than to their cholesterol content, the broader and more heterogeneous nature of non-HDL-C may partly explain the weaker observed association with specific PON1 activity [15].

Nevertheless, the relatively modest adjusted R^2^ values indicate that specific PON1 activity explains only a limited proportion of the variability in atherogenic lipid measures. This suggests that additional biological and environmental determinants contribute to both lipid profiles and oxidative status, and that these associations should be interpreted with appropriate caution. Taken together, these findings extend current knowledge by suggesting that specific PON1 activity is associated with selected lipid measures of atherogenic lipoproteins and some conventional lipid parameters, supporting further investigation of combined HDL functional and quantitative lipid metrics.

These findings may have potential clinical implications for cardiovascular risk assessment. Measuring specific PON1 activity in HDL-associated fractions that preserve the enzyme’s native environment may represent a functionally informative research measure. Such an approach could be investigated as a marker of altered HDL-associated antioxidant function and may help refine residual cardiometabolic risk assessment in future validated studies. However, assay standardization and longitudinal validation are required before PON1-related measures can be considered for routine clinical use.

Finally, this study has several limitations. First, its cross-sectional design does not allow for drawing conclusions about relationships between specific PON1 activity and lipid metabolism measures over time. Second, the participants were recruited consecutively from a single cardiovascular prevention center rather than through population-based random sampling, the possibility of selection bias cannot be excluded. Third, the study population consisted of middle-aged individuals at elevated cardiovascular risk, restricting generalizability to healthy populations or to populations of varying ages. Given the relatively modest sample size (n = 100) and the number of regression models and subgroup-style tertile analyses performed, the study may have had limited statistical power to detect small associations. Therefore, the findings should be interpreted as exploratory and hypothesis-generating rather than definitive. To reduce the risk of false-positive results arising from multiple comparisons, false discovery rate (FDR) correction was applied where appropriate. The absence of a control group further limits the ability to compare with a healthy reference population. In addition, information on medication use beyond statin therapy was unavailable. Therefore, residual confounding related to other treatments that may affect lipid metabolism or PON1 activity cannot be ruled out. Methodologically, even though iodixanol-based density gradient centrifugation preserves lipoprotein integrity, it provides limited resolution, and the analyzed fractions do not represent pure HDL subfractions. Finally, despite multivariable adjustment, residual confounding cannot be ruled out due to unmeasured factors, such as diet, lifestyle, other medication use, PON1 genetic polymorphisms, oxidative stress, and inflammatory status.

## Figures and Tables

**Table 1:** Overall sample characteristics.

Variable	Value (n = 100)
Demographic and anthropometric measures	
Age (years)	52.0 (9.5)
Female sex, n (%)	50 (50.0)
BMI (kg/m^2^)	28.76 (7.63)
Waist circumference (cm)	101.65 ± 13.84
Blood pressure measures	
SBP (mmHg)	137.00 (22.25)
DBP (mmHg)	84.14 ± 10.57
MABP (mmHg)	101.17 (16.83)
Lipid metabolism measures	
Total cholesterol (mmol/L)	5.73 ± 1.27
LDL-C (mmol/L)	3.61 ± 1.18
HDL-C (mmol/L)	1.34 (0.45)
Non-HDL-C (mmol/L)	4.31 ± 1.22
Triglycerides (mmol/L)	1.35 (0.89)
Apo B (g/L)	0.95 ± 0.25
Apo A-I (g/L)	1.65 (0.41)
Apo B/Apo A-I	0.57 ± 0.18
Lp(a) (nmol/L)	18.45 (36.45)
Rw Apo B (µmol/L)	2.11 (0.75)
PON1-related measures	
PON1 (ng/mL)	8.69 (11.48)
PON1 activity (U/mL)	0.08 ± 0.03
Other characteristics	
Smoking status, n (%)	50 (50.0)
Statin use, n (%)	38 (38.0)
Hypertension, n (%)	57 (57.0)
Diabetes mellitus, n (%)	13 (13.0)
Metabolic Syndrome, n (%)	50 (50.0)
Family history of CVD, n (%)	40 (40.0)

Data are presented as mean ± SD, median (IQR), or n (%), as appropriate. Abbreviations: BMI, body mass index; SBP, systolic blood pressure; DBP, diastolic blood pressure; MABP, mean arterial blood pressure; LDL-C, low-density lipoprotein cholesterol; HDL-C, high-density lipoprotein cholesterol; Non-HDL-C, non-high-density lipoprotein cholesterol; Apo B, apolipoprotein B; Apo A-I, apolipoprotein A-I; Apo B/Apo A-I, apolipoprotein B to apolipoprotein A-I ratio; Lp(a), lipoprotein(a); Rw Apo B, risk-weighted apolipoprotein B; PON1, paraoxonase 1; PON1 activity, paraoxonase 1 activity, CVD, cardiovascular disease.

**Table 2:** Sample characteristics stratified by tertiles of specific PON1 activity.

Variable	T1 (n = 34) *	T2 (n = 33) *	T3 (n = 33) *	*p*-Value	q-Value	p-for Trend
Demographic and anthropometric measures						
Age (years)	52.0 (9.5)	54.0 (6.0)	52.0 (10.0)	0.569	0.642	0.363
Female sex, n (%)	17 (50.0)	14 (42.4)	19 (57.6)	0.469	0.642	-
BMI (kg/m^2^)	28.86 (6.70)	29.40 (8.50)	27.94 (7.60)	0.390	0.642	0.716
Waist circumference (cm)	102.03 ± 13.32	104.52 ± 12.99	98.34 ± 14.89	0.214	-	0.288
Blood pressure measures						
SBP (mmHg)	139.00 (21.50)	137.00 (14.00)	135.00 (29.00)	0.652	0.642	0.185
DBP (mmHg)	84.03 ± 12.18	85.79 ± 9.70	82.61 ± 9.66	0.418	-	0.591
MABP (mmHg)	101.00 (18.83)	103.33 (9.00)	96.67 (15.67)	0.478	0.642	0.327
Lipid metabolism measures						
Total cholesterol (mmol/L)	5.94 ± 1.25	5.58 ± 1.36	5.65 ± 1.21	0.466	-	0.340
LDL-C (mmol/L)	3.97 ± 1.07	3.46 ± 1.22	3.41 ± 1.19	0.081	-	0.048
HDL-C (mmol/L)	1.31 (0.43)	1.31 (0.35)	1.42 (0.37)	0.282	0.642	0.094
Non-HDL-C (mmol/L)	4.61 ± 1.08	4.18 ± 1.35	4.15 ± 1.22	0.199	-	0.127
Triglycerides (mmol/L)	1.29 (0.64)	1.38 (0.99)	1.36 (0.96)	0.700	0.642	0.182
Apo B (g/L)	1.00 ± 0.22	0.93 ± 0.30	0.91 ± 0.23	0.258	-	0.152
Apo A-I (g/L)	1.61 (0.48)	1.66 (0.37)	1.77 (0.46)	0.323	0.642	0.114
Apo B/Apo A-I	0.62 ± 0.15	0.56 ± 0.20	0.53 ± 0.17	0.101	-	0.052
Lp(a) (nmol/L)	25.95 (101.70)	15.00 (24.20)	13.50 (33.80)	0.095	0.348	0.051
Rw Apo B (µmol/L)	2.31(0.82)	2.02 (0.70)	2.03 (0.69)	0.023	0.127	0.015
PON1-related measures						
PON1 (ng/mL)	25.10 (30.99)	8.80 (5.52)	5.46 (1.63)	<0.001	<0.001	<0.001
PON1 activity (U/mL)	0.05 ± 0.03	0.08 ± 0.02	0.10 ± 0.02	<0.001	-	<0.001
Other characteristics						
Smoking status, n (%)	17 (50.0)	15 (45.5)	18 (54.5)	0.761	0.761	-
Statin use, n (%)	11 (32.4)	15 (45.5)	12 (36.4)	0.528	0.676	-
Hypertension, n (%)	17 (50.0)	21 (63.6)	19 (57.6)	0.528	0.676	-
Diabetes mellitus, n (%)	2 (5.9)	6 (18.2)	5 (15.2)	0.295	0.676	-
Metabolic Syndrome, n (%)	16 (47.1)	19 (57.6)	15 (45.5)	0.563	0.676	-
Family history of CVD, n (%)	8 (23.5)	16 (48.5)	16 (48.5)	0.054	0.324	-

Data are presented as mean ± SD, median (IQR), or n (%), as appropriate. * Specific paraoxonase 1 activity tertiles were defined as T1 ≤ 5.68 kU/mg, T2 5.68- 12.45 kU/mg and T3 ≥ 12.45 kU/mg. Abbreviations: BMI, body mass index; SBP, systolic blood pressure; DBP, diastolic blood pressure; MABP, mean arterial blood pressure; LDL-C, low-density lipoprotein cholesterol; HDL-C, high-density lipoprotein cholesterol; Non-HDL-C, non-high-density lipoprotein cholesterol; Apo B, apolipoprotein B; Apo A-I, apolipoprotein A-I; Apo B/Apo A-I, apolipoprotein B to apolipoprotein A-I ratio; Lp(a), lipoprotein(a); Rw Apo B, risk-weighted apolipoprotein B; PON1, paraoxonase 1; PON1 activity, paraoxonase 1 activity; Specific PON1 activity, paraoxonase 1 activity normalized to paraoxonase 1 protein concentration, CVD, Cardiovascular Disease.

**Table 3:** Associations between specific PON1 activity and demographic, anthropometric, and blood pressure measures.

Variable	Spearman r_s_ with Specific PON1 Activity (U/mg)	*p*-Value	q-Value
Demographic and anthropometric measures			
Age (years)	−0.103	0.310	0.680
BMI (kg/m^2^)	−0.061	0.548	0.658
Waist circumference (cm)	−0.133	0.189	0.680
Blood pressure measures			
SBP (mmHg)	−0.089	0.377	0.680
DBP (mmHg)	−0.042	0.680	0.680
MABP (mmHg)	−0.070	0.491	0.680

Abbreviations: BMI, body mass index; SBP, systolic blood pressure; DBP, diastolic blood pressure; MABP, mean arterial blood pressure; Specific PON1 activity, paraoxonase 1 activity normalized to paraoxonase 1 protein concentration.

**Table 4:** Associations between specific PON1 activity and lipid metabolism measures.

Variable	Spearman r_s_ with Specific PON1 Activity (kU/mg)	*p*-Value	q-Value
Lipid metabolism measures			
Total cholesterol (mmol/L)	−0.120	0.235	0.261
LDL-C (mmol/L)	−0.225	0.025	0.083
HDL-C (mmol/L)	0.139	0.167	0.209
Non-HDL-C (mmol/L)	−0.165	0.100	0.167
Triglycerides (mmol/L)	0.023	0.820	0.820
Apo B (g/L)	−0.189	0.059	0.118
Apo A-I (g/L)	0.152	0.132	0.189
Apo B/Apo A-I	−0.240	0.016	0.209
Lp(a) (nmol/L)	−0.192	0.045	0.113
Rw Apo B (nmol/L)	−0.287	0.004	0.040

Abbreviations: LDL-C, low-density lipoprotein cholesterol; HDL-C, high-density lipoprotein cholesterol; Non-HDL-C, non-high-density lipoprotein cholesterol; Apo B, apolipoprotein B; Apo A-I, apolipoprotein A-I; Apo B/Apo A-I, apolipoprotein B to apolipoprotein A-I ratio; Lp(a), lipoprotein(a); Rw Apo B, risk-weighted apolipoprotein B; Specific PON1 activity, paraoxonase 1 activity normalized to paraoxonase 1 protein concentration.

**Table 5:** Multivariable regression results for atherogenic lipid metabolism parameters.

Dependent Variable	Model *	β [95% CI] ***	Adjusted R^2^	*p*-Value	q-Value
Apo B (g/L)	Model 1	−0.007 [−0.014; 0.000]	0.025	0.064	0.069
	Model 2	−0.007 [−0.014; 0.000]	0.042	0.052	0.066
	Model 3	−0.007 [−0.014; 0.000]	0.038	0.063	0.069
	Model 4	−0.006 [−0.013; 0.001]	0.115	0.086	0.086
Apo B/Apo A-I	Model 1	−0.006 [−0.011; −0.001]	0.043	0.021	0.043
	Model 2	−0.005 [−0.010; −0.001]	0.163	0.023	0.043
	Model 3	−0.005 [−0.009; −0.000]	0.205	0.036	0.058
	Model 4	−0.004 [−0.009; 0.000]	0.238	0.051	0.066
ln (Rw Apo B)	Model 1	−0.013 [−0.022; −0.004]	0.071	0.004	0.032
	Model 2	−0.014 [−0.022; −0.005]	0.075	0.003	0.032
	Model 3	−0.013 [−0.022; −0.004]	0.067	0.004	0.032
	Model 4	−0.012 [−0.021; −0.004]	0.101	0.006	0.034
LDL-C (mmol/L)	Model 1	−0.038 [−0.071; −0.006]	0.053	0.021	0.043
	Model 2	−0.042 [−0.074; −0.009]	0.082	0.013	0.033
	Model 3	−0.045 [−0.077; −0.012]	0.110	0.007	0.034
	Model 4	−0.041 [−0.071; −0.010]	0.230	0.010	0.040
Non-HDL-C (mmol/L)	Model 1	−0.032 [−0.066; 0.002]	0.024	0.066	0.069
	Model 2	−0.035 [−0.069; −0.001]	0.040	0.045	0.064
	Model 3	−0.035 [−0.070; −0.001]	0.030	0.045	0.064
	Model 4	−0.031 [−0.063; 0.002]	0.146	0.064	0.079
ln (Lp(a)) **	Model 1	−0.039 [−0.074; −0.003]	0.032	0.034	0.058
	Model 2	−0.043 [−0.078; −0.008]	0.040	0.016	0.043
	Model 3	−0.044 [−0.079; −0.009]	0.032	0.015	0.043
	Model 4	−0.042 [−0.078; −0.006]	0.030	0.023	0.043

* Model 1—unadjusted model with only specific PON1 activity, Model 2—adjusted for age and sex, Model 3—additionally adjusted for BMI, Model 4—additionally adjusted for statin use and smoking status. ** ln (Lp(a)) Robust standard errors (HC3) were used. Abbreviations: LDL-C, low-density lipoprotein cholesterol; Non-HDL-C, non-high-density lipoprotein cholesterol; Apo B, apolipoprotein B; Apo B/Apo A-I, apolipoprotein B to apolipoprotein A-I ratio; ln (Lp(a)), natural logarithm of lipoprotein(a); ln (Rw Apo B), natural logarithm of risk-weighted apolipoprotein B. *** β coefficients represent the association with specific PON1 activity per 1 kU/mg increase.

## Data Availability

The datasets presented in this article are not readily available due to ethical considerations and the need to protect the privacy and confidentiality of study participants. Requests to access the datasets should be directed to the corresponding author.

## Supplementary Materials

The following supporting information can be downloaded at: https://www.mdpi.com/article/10.3390/antiox15060731/s1, Table S1: Multivariable regression assumption check results and model statistical significance values; Table S2: Sensitivity analysis for dependent variables LDL-C and ln (Rw Apo B).

## Abbreviations

The following abbreviations are used in this manuscript:

Apo A-I	Apolipoprotein A-I
Apo B	Apolipoprotein B
Apo B/Apo A-I	Apolipoprotein B and apolipoprotein A-I ratio
BMI	Body Mass Index
CVD	Cardiovascular disease
DBP	Diastolic Blood Pressure
DGC	Density Gradient Centrifugation
FDR	False Discovery Rate
HDL	High-density lipoprotein
HDL-C	High-density lipoprotein cholesterol
LDL-C	Low-density lipoprotein cholesterol
Lp(a)	Lipoprotein(a)
MABP	Mean Arterial Blood Pressure
Non-HDL-C	Non-High-density lipoprotein cholesterol
pNP	p-nitrophenol
pNPA	p-nitrophenyl acetate
PON1	Paraoxonase 1
Rw Apo B	Risk-weighted Apolipoprotein B
SBP	Systolic Blood Pressure
TE	Tris-EDTA
TG	Triglycerides
