# Supplementary material for: HDL-Associated Specific Paraoxonase-1 Activity Is Linked to Atherogenic Lipoprotein Measures in a High Cardiovascular Risk Population: A Cross-Sectional Study
Source: Antioxidants (Basel). 2026 Jun 9;15(6):731. doi: 10.3390/antiox15060731 (PMC13295251; doi:10.3390/antiox15060731)
Supplement: Supplementary file 1 [file antioxidants-15-00731-s001.zip › antioxidants-4327319-supplementary.pdf]

Table S1. Multivariable regression assumption check results and model statistical significance values.

Dependent Variable	Model *	Linearity (Visual)	Normality of Residuals (Shapiro-Wilk)	Homoscedasticity (Breusch-Pagan)	VIF	Influential Points (Cook's D, Max)	Assumptions Met	F-Test p-Value
Apo B (g/L)	Model 1	No pattern	0.447	0.650	1.00	0.168	Yes	0.064
	Model 2	No pattern	0.394	0.788	1.02	0.104	Yes	0.068
	Model 3	No pattern	0.236	0.831	1.03	0.084	Yes	0.105
	Model 4	No pattern	0.326	0.985	1.23	0.072	Yes	0.008
Apo B/Apo A-I	Model 1	No pattern	0.321	0.915	1.00	0.103	Yes	0.021
	Model 2	No pattern	0.181	0.643	1.02	0.094	Yes	<0.001
	Model 3	No pattern	0.984	0.512	1.03	0.082	Yes	<0.001
	Model 4	No pattern	0.335	0.773	1.23	0.093	Yes	<0.001
ln (Rw Apo B)	Model 1	No pattern	0.842	0.785	1.00	0.051	Yes	0.004
	Model 2	No pattern	0.925	0.364	1.02	0.090	Yes	0.015
	Model 3	No pattern	0.933	0.462	1.03	0.081	Yes	0.032
	Model 4	No pattern	0.546	0.421	1.23	0.120	Yes	0.013
	Model 4 + Hypertension	No pattern	0.145	0.672	1.27	0.136	Yes	0.013
	Model 4 + Metabolic Syndrome	No pattern	0.681	0.200	1.37	0.112	Yes	0.001
LDL-C (mmol/L)	Model 1	No pattern	0.447	0.650	1.00	0.168	Yes	0.021
	Model 2	No pattern	0.394	0.788	1.02	0.104	Yes	0.042
	Model 3	No pattern	0.236	0.831	1.03	0.084	Yes	0.024
	Model 4	No pattern	0.326	0.986	1.23	0.072	Yes	<0.001
	Model 4 + Hypertension	No pattern	0.070	0.976	1.28	0.094	Yes	<0.001
	Model 4 + Metabolic Syndrome	No pattern	0.287	0.940	1.37	0.081	Yes	<0.001
Non-HDL-C (mmol/L)	Model 1	No pattern	0.523	0.597	1.00	0.152	Yes	0.066
	Model 2	No pattern	0.602	0.899	1.02	0.098	Yes	0.074
	Model 3	No pattern	0.562	0.960	1.03	0.078	Yes	0.140

	Model 4	No pattern	0.126	0.968	1.23	0.065	Yes	0.002
ln [Lp(a)] **	Model 1	No pattern	0.002	0.179	1.00	0.054	Partially	0.042
	Model 2	No pattern	0.002	0.329	1.02	0.076	Partially	0.076
	Model 3	No pattern	0.003	0.375	1.03	0.060	Partially	0.132
	Model 4	No pattern	0.008	0.399	1.23	0.062	Partially	0.186

* Model 1 - unadjusted model with only specific PON1 activity, Model 2 - adjusted for age and sex, Model 3 - additionally adjusted for BMI, Model 4 - additionally adjusted for statin use and smoking status. ** For ln[Lp(a)] models, HC3 robust standard errors were used due to the non-normality of residuals. Abbreviations: LDL-C, low-density lipoprotein cholesterol; Non-HDL-C, non-high-density lipoprotein cholesterol; Apo B, apolipoprotein B; Apo B/Apo A-I, apolipoprotein B to apolipoprotein A-I ratio; ln [Lp(a)], natural logarithm of lipoprotein(a); ln (Rw Apo B), natural logarithm of risk-weighted apolipoprotein B, VIF - Variance Inflation Factor.

Table S2. Sensitivity analysis for dependent variables LDL-C and ln (Rw Apo B).

**Dependent Variable**	**Model ***	**β [95% CI] ****	**Adjusted R^2**	**p-Value**	**q-Value**
ln (Rw Apo B)	Model 4 + Hypertension	-0.012 [-0.020; -0.003]	0.171	**0.011**	**0.014**
	Model 4 + Metabolic Syndrome	-0.013 [-0.021; -0.004]	0.217	**0.004**	**0.014**
LDL-C (mmol/L)	Model 4 + Hypertension	-0.035 [-0.065; -0.005]	0.280	**0.014**	**0.014**
	Model 4 + Metabolic Syndrome	-0.041 [-0.072; -0.010]	0.233	**0.010**	**0.014**

* Model 1—unadjusted model with only specific PON1 activity, Model 2—adjusted for age and sex, Model 3—additionally adjusted for BMI, Model 4—additionally adjusted for statin use and smoking status. ** β coefficients represent the association with specific PON1 activity per 1 kU/mg increase.
